# Accuracy assessment of patient safety incident (PSI) codes and present-on-admission (POA) indicators: a cross-sectional analysis using the Patient Safety Incidents Inquiry (PSII) in Korea

**DOI:** 10.1186/s12913-024-11210-w

**Published:** 2024-06-21

**Authors:** Jeehee Pyo, Eun Young Choi, Seung Gyeong Jang, Won Lee, Minsu Ock

**Affiliations:** 1 Department of Preventive Medicine, Ulsan University Hospital, University of Ulsan College of Medicine, 25 Daehagbyeongwon-Ro, Dong-Gu, Ulsan, 44033 Republic of Korea; 2 Always Be With You, The PLOCC Affiliated Counseling Training Center, Seoul, Republic of Korea; 3 Department of Nursing, Chung-Ang University, 84 Heukseok-Ro, Dongjak-Gu, Seoul, 06974 Republic of Korea; 4 Department of Nursing, Inje University, Busan, Republic of Korea; 5 Department of Preventive Medicine, University of Ulsan College of Medicine, Seoul, Republic of Korea

**Keywords:** Patient safety, Present-on-admission, International Classification of Diseases (ICD), Medical record review

## Abstract

**Background:**

Among the various methods used, administrative data collected for claims and billing purposes, such as diagnosis codes and present-on-admission (POA) indicators, can easily be employed to assess patient safety status. However, it is crucial that administrative data be accurate to generate valid estimates of adverse event (AE) occurrence. Thus, we aimed to evaluate the accuracy of diagnosis codes and POA indicators in patients with confirmed AEs in the hospital admission setting.

**Methods:**

We analysed the diagnosis codes of 1,032 confirmed AE cases and 6,754 non-AE cases from the 2019 Patient Safety Incidents Inquiry, which was designed as a cross-sectional study, to determine their alignment with the Korean Patient Safety Incidents (PSIs) Code Classification System. The unit of analysis was the individual case rather than the patient, because two or more AEs may occur in one patient. We examined whether the primary and secondary diagnostic codes had PSIs codes matching the AE type and checked each PSI code for whether the POA indicator had an ‘N’ tag. We reviewed the presence of PSI codes in patients without identified AEs and calculated the correlation between the AE incidence rate and PSI code and POA indicator accuracy across 15 hospitals.

**Results:**

Ninety (8.7%) of the AE cases had PSI codes with an ‘N’ tag on the POA indicator compared to 294 (4.4%) of the non-AE cases. Infection- (20.4%) and surgery/procedure-related AEs (13.6%) had relatively higher instances of correctly tagged PSI codes. We did not identify any PSI codes for diagnosis-related incidents. While we noted significant differences in AE incidence rates, PSI code accuracy, and POA indicator accuracy among the hospitals, the correlations between these variables were not statistically significant.

**Conclusion:**

Currently, PSI codes and POA indicators in South Korea appear to have low validity. To use administrative data in medical quality improvement activities such as monitoring patient safety levels, improving the accuracy of administrative data should be a priority. Possible strategies include targeted education on PSI codes and POA indicators and introduction of new evaluation indicators regarding the accuracy of administrative data.

## Background

Among the diverse methods used to identify patient safety incidents (PSIs), administrative data have been increasing use [1–4]. Administrative data such as claims and billing information are amassed through numerous channels during medical processes and stand out for their relative ease of use compared to other kinds of data or methods used to assess patient safety status [5]. Furthermore, this approach capitalises on already-collected data, thereby eliminating the need for additional expenditure on data collection, facilitating efficient evaluation of patient safety status [5]. Diagnosis codes are a form of administrative data extensively utilised to determine disease burden, as measured by indicators such as disability-adjusted life years, and ensure a high level of comparability in the outcomes derived [6, 7].

In this context, attempts have been made to develop a PSI code classification system aimed at systematically and comprehensively gathering data on PSI cases via diagnosis codes [8, 9]. For instance, Choi et al. [9] classified a total of 4,509 codes from the Korean Standard Classification of Disease, Version 7 (KCD-7) into six types of PSIs. However, to distinguish the occurrence or prevalence of PSIs, a metric known as present-on-admission (POA) is needed in addition to PSI-related diagnosis codes. A POA indicator further annotates a diagnosis code to clarify whether the patient’s condition was present before admission (‘Y’-tagged) or if it developed after admission (‘N’-tagged) [10]. Thus, examining both PSI codes and POA indicators is crucial for identifying PSIs that transpired during a hospital stay from those that did not.

To effectively monitor the occurrence of PSIs using PSI codes and POA indicators, it is vital to ensure the accuracy of both metrics. For instance, research comparing complication data from Canada’s National Surgical Quality Improvement Program with PSI codes highlighted the insufficiency of PSI code accuracy for individual-level patient safety monitoring [11]. Likewise, a study assessing complication data against South Korea’s healthcare quality improvement checklist indicated a need to enhance the accuracy of both PSI codes and POA indicators [12].

However, there is a significant research gap in terms of concurrently examining the accuracy of PSI codes and POA indicators. Most previous studies have focused solely on assessing the accuracy of POA indicators for specific PSI types [13–15]. Even research evaluating the accuracy of both PSI codes and POA indicators has faced limitations by relying on another set of administrative data as a benchmark [12]. To address this research gap, we conducted an in-depth analysis of PSI code and POA indicator entries at the national level based on the Patient Safety Incidents Inquiry (PSII) [16, 17], which is conducted in South Korea. We attempted to evaluate the accuracy of PSI codes and POA indicators from multiple perspectives across participating medical institutions in PSII.

## Methods

### Data sources

#### Adverse events, diagnosis codes, and *POA* indicators

We utilised data on adverse events (AEs), diagnosis codes, and POA indicators collected through the PSII [16, 17]. The PSII involves a comprehensive review of medical records of 500 patients discharged from 15 regional public hospitals each across South Korea, totalling 7,500 patients. The PSII was implemented in 2019 and involved a three-stage retrospective review of medical records for patients discharged in 2016 from regional public hospitals dedicated to acute care. This review process identified 1,032 cases of AEs, which were classified into six distinct categories [16].

The PSII collected information by having two reviewers independently review medical records at each review stage [16, 17]. We included diagnosis codes documented by at least one of the two first reviewers of the PSII in the analysis, which incorporated both the primary and secondary diagnostic codes of the patients. As with diagnosis codes, if even one first reviewer tagged the POA indicator as ‘N’, we considered the POA indicator for the diagnosis code to be ‘N’.

#### PSI code classification

We identified PSI codes using the Korean Patient Safety Incidents Code Classification System [9]. This system was developed by reviewing and categorising the KCD-7 codes using the Delphi method. Of the 54,550 KCD-7 codes, 4,509 were identified as PSI codes and classified into six major categories, consistent with the types of AEs established in the PSII.

### Data analysis

#### Accuracy of PSI codes and *POA* indicators for AEs

The unit of analysis was the individual case rather than the patient, because two or more AEs may occur in one patient. We assessed the accuracy of the PSI codes by verifying the presence of PSI codes corresponding to the six types of AEs identified in the PSII within the patients’ diagnosis codes. For instance, if an AE was classified as an issue related to patient care (e.g. pressure ulcers) in the PSII, we checked whether any of the patient’s diagnosis codes had been identified as patient care-related PSI codes (e.g. for pressure ulcers, whether any of L89.0, L89.1, L89.2, L89.3, or L89.9 was present) within the Korean Patient Safety Incidents Code Classification System. As the PSII identifies AEs that occurred during hospital admission, if a PSI code exists, the POA indicator tagged with it should be ‘N’.

**Fig. 1:**
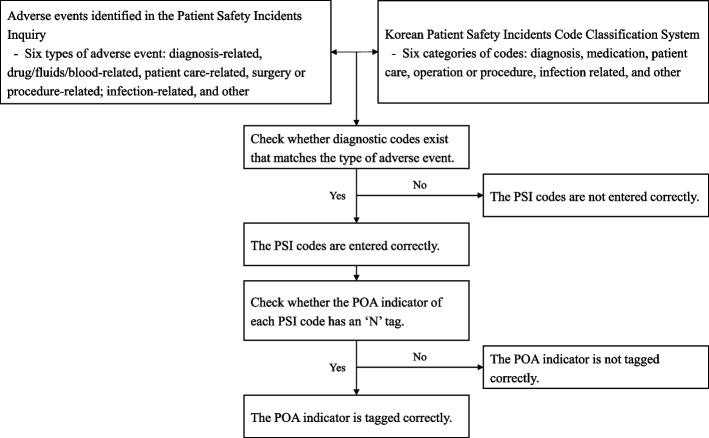
Analysis framework to assess the accuracy of PSI codes and POA indicators for AEs. Note: AEs: adverse events; PSI: patient safety incident; POA: present-on-admission

We determined the accuracy of the POA indicator of each PSI code, classified by AE type, based on whether it was tagged with ‘N’. We calculated the presence and absence of PSI codes by AE type and the ‘N’ tag for POA indicators in terms of frequency (n) and percentage (%). This process is illustrated in Fig. 1.

#### Sensitivity and specificity

For non-AE cases, we checked whether the diagnosis codes included PSI codes irrespective of AE type, and if so, whether those PSI codes had an ‘N’-tagged POA indicator. We calculated sensitivity as the percentage of cases identified as AEs that had PSI codes with a POA indicator tagged as ‘N’, and we calculated specificity as the percentage of non-AE cases that did not have a PSI code with a POA indicator tagged as ‘N’. We calculated both values (%) and their 95% confidence intervals (CIs).

#### Analysis by hospital

For the analysis by hospital, we first calculated the frequency (n) and percentage (%) of AE cases for each hospital. Then, we applied the aforementioned analysis method for the accuracy of PSI codes and POA indicators to establish their accuracy for each hospital. Finally, we analysed the correlation between these variables using Pearson’s correlation method.

#### Statistical analysis

We sorted and analysed the data using Microsoft Excel and SPSS (version 25.0).

## Results

### Accuracy of PSI codes with *POA* indicators tagged as ‘N’ for AEs

Table 1. outlines the distribution of PSI codes with POA indicators tagged as ‘N’ based on the presence or absence of AEs. Of the 1,032 identified AE cases, 90 (8.7%) were assigned PSI codes with POA indicators tagged as ‘N’ that corresponded to the type of AE. Among the 6,754 non-AE cases, 294 (4.4%) were assigned PSI codes with POA indicators tagged as ‘N’; the remaining 6,460 cases (95.6%) either lacked PSI codes or had PSI codes tagged with values other than ‘N’ for a POA indicator. The sensitivity of utilising PSI codes with POA indicators tagged as ‘N’ to confirm AEs was 8.7% (95% CI: 7.1%–10.5%), while the specificity was 95.1% (95% CI: 94.1%–96.1%).

**Table 1.** Accuracy of PSI codes with POA indicators tagged as ‘N’ relative to AEs

		PSI codes with POA indicators tagged as ‘N’			
		Present		Absent	
		N	% of row	N	% of row
AE	Yes (*n* = 1,032)	90^a^	8.7	942	91.3
	No (*n* = 6,754)	294	4.4	6,460	95.6
	Total (*n* = 7,786)	384	4.9	7,402	95.1

*AE* Adverse event, *POA* Present-on-admission, *PSI* Patient safety incident

^a^PSI codes correspond to the types of AEs

### Accuracy of PSI codes and *POA* indicators by type of AE

Table 2. presents the analysis results of PSI codes and POA indicators by AE type. The most common type of AE was issues related to patient care (*n* = 346, 33.5%) followed by medication, fluids, and blood (*n* = 268, 26.0%). Of all 1,032 AE cases, 346 (33.5%) had PSI codes corresponding to their AE type with 90 of these (26.0%) providing ‘N’-tagged POA indicators. A closer look by AE type revealed that the type for which the PSI codes were most accurately assigned was infections (56.8%), followed by patient care (34.4%) and surgery or procedures (31.5%). Surgery or procedure-related AEs (43.1%) had the highest proportion of ‘N’-tagged POA indicators, followed by AEs related to infection (35.9%) and medication, fluids, and blood (24.7%). No PSI codes were assigned to diagnosis-related AEs.

**Table 2.** Accuracy of PSI codes and POA indicators by type of AE

Types of AEs	PSI codes^a^ present		PSI codes^a^ with POA indicator tagged as ‘N’ present		
	N	% of AEs	N	% of AEs	% of PSI codes*
1) Diagnosis-related (*n* = 72)	0	0.0	0	0.0	0.0
2) Drug/Fluids/Blood-related (*n* = 268)	77	28.7	19	7.1	24.7
3) Patient care-related (*n* = 346)	119	34.4	13	3.8	10.9
4) Surgery or procedure-related (*n* = 184)	58	31.5	25	13.6	43.1
5) Infection-related (*n* = 162)	92	56.8	33	20.4	35.9
Total (*n* = 1,032)	346	33.5	90	8.7	26.0

*AE* Adverse event, *POA* Present-on-admission, *PSI* Patient safety incident

^a^PSI codes correspond to the types of AEs

### Accuracy of PSI codes and *POA* indicators by hospital

Table 3. outlines the results of analysing AE incidence rates and the accuracy of PSI codes and POA indicators across the 15 hospitals. The incidence rate of AEs showed a substantial variation of 36.6% across the institutions (range: 1.2%–37.8%). Similarly, the accuracy of PSI codes related to AE type exhibited a disparity of 51.8% (range: 13.4%–65.2%), and the proportion of PSI codes with POA indicators tagged as ‘N’ varied by 64.6% (range: 0.0%–64.6%). The statistical analysis revealed no significant correlations among all three variable pairs across the hospitals: AE incidence rate and accuracy of the PSI codes (*r* =  − 0.061, *P*-value = 0.829); AE incidence rate and accuracy of PSI codes with POA indicators tagged as ‘N’ (*r* =  − 0.103, *P*-value = 0.715); and accuracy of PSI codes with POA indicators tagged as ‘N’ (*r* = 0.332, *P*-value = 0.226).

**Table 3.** Accuracy of AE incidence rates, PSI codes, and POA indicators of each hospital

Hospital	Reviews	AEs		PSI codes (present)		PSI codes with POA indicators tagged as ‘N’ present	
	N	N	% of reviews	N	% of AEs	N	% of PSI codes
A	504	64	12.7	18	28.1	5	27.8
B	503	10	2.0	4	40.0	0	0.0
C	519	84	16.2	25	29.8	8	32.0
D	629	238	37.8	102	42.9	4	3.9
E	500	6	1.2	2	33.3	0	0.0
F	507	69	13.6	12	17.4	3	25.0
G	521	94	18.0	21	22.3	8	38.1
H	539	111	20.6	43	38.7	13	30.2
I	512	66	12.9	34	51.5	23	67.6
J	505	23	4.6	15	65.2	8	53.3
K	517	82	15.9	11	13.4	1	9.1
L	516	70	13.6	29	41.4	12	41.4
M	507	61	12.0	16	26.2	0	0.0
N	503	33	6.6	7	21.2	1	14.3
O	504	21	4.2	7	33.3	4	57.1
Total	7786	1032	13.3	346	33.3	90	26.0

*AE* Adverse event, *POA* Present-on-admission, *PSI* Patient safety incident

## Discussion

In this study, we analysed the diagnosis codes and POA indicators of patients with confirmed AEs based on data from the PSII to evaluate the accuracy of PSI codes and POA indicators. Our methodology involved estimating the accuracy of PSI codes and POA indicators by verifying the presence of PSI codes corresponding to AE type, identified in patients’ diagnosis codes and whether the POA indicators assigned to these PSI codes were tagged as ‘N’. The evaluation results indicated low accuracy of PSI codes and POA indicators in South Korean hospitals. Analysis of each hospital’s data showed considerable variation in AE incidence rates and the accuracy of PSI codes and POA indicators. This study’s primary contribution is in highlighting the critical need to enhance the accuracy of diagnosis codes and POA indicators in administrative data as a means to more effectively gauge patient safety status.

Among the methods used to assess patient safety status, administrative data (including diagnosis codes) has commonly been used to detect the presence of various types of PSIs [1–4]. However, the comprehensive accuracy of this approach remains underexplored with most studies focusing solely on the accuracy of POA indicators for certain PSI categories [13–15]. The only known study that concurrently assessed a range of PSI codes and POA indicators has limited precision in evaluating accuracy due to its reliance on cross-validation of administrative data [12]. For a more robust evaluation of PSI codes and POA indicators’ accuracy, it is imperative to compare these findings with those obtained from methodologies deemed the gold standard in patient safety assessment, such as the medical records review [18]. This study is unique in that it validates the analysis method against the PSII, which verifies AE occurrences through a review of medical records, thereby serving as a comparative benchmark and enhancing the study’s validity [16].

Moreover, this study stands out for its comprehensive evaluation of the accuracy of PSI codes and POA indicators across six types of AEs, encompassing diagnosis-related AEs and facilitating a comparison of accuracy across different PSI types. With the overall average accuracy of PSI codes with POA indicators tagged as ‘N’ at 8.7%, we observed lower accuracy rates in AEs related to diagnosis, medication/fluids/blood, and patient care. We noted higher accuracy rates in AEs related to surgery/procedure and infection. The absence of PSI codes in diagnosis-related AEs might be understandable given the limited number of PSI codes—only two—available in the Korean Patient Safety Incidents Code Classification System [9]. Nonetheless, the lack of codes for patient care-related AEs (such as pressure ulcers)—despite the presence of specific codes like L890 (Stage I decubitus ulcer and pressure area) and L891 (Stage II decubitus ulcer and pressure area)—highlights significant oversight. These findings suggest that using pressure ulcer-related PSI codes to infer a decline in the incidence of pressure ulcers in South Korea may not be a valid approach [19].

Our findings indicate that the absence of diagnosis codes in administrative data is not limited to specific types of PSIs (such as pressure ulcers). Even in cases of infection-related AEs—which demonstrate comparatively high accuracy for PSI codes and POA indicators—AE-related accuracy remains alarmingly low, ranging from 20 to 30%. This underscores the urgent need for comprehensive interventions in South Korean hospitals to improve the accuracy of PSI codes and POA indicators within their administrative databases. Most importantly, there is a need to raise the awareness level of healthcare professionals, especially physicians, regarding PSI codes and POA indicators [20].

In South Korea, although health information managers play a supportive role in coding tasks, the primary responsibility and authority for code assignment rests with physicians. Thus, code assignment should be prioritised to monitor physicians’ level of awareness and improve their familiarity with PSI codes and POA indicators. At the same time, it is imperative to establish detailed training and guidelines for accurately entering PSI codes and POA indicators. While South Korea has somewhat established guidelines and training for POA indicators [21], such resources for PSI codes are notably absent. Given the diminished effectiveness of POA indicators without accurate PSI codes entered, developing targeted guidelines and training programmes for PSI codes should be a top priority.

Given the notable variability in the accuracy of PSI codes and POA indicators across South Korean hospitals, it may be beneficial for hospitals to adopt diverse evaluation metrics for assessing the precision of PSI codes and POA indicators [22]. At the most basic level, the presence of staff specifically tasked with managing PSI codes and POA indicators should be checked, including their completion of necessary training. Additionally, whether the hospitals conduct their own audits to improve the accuracy of PSI codes and POA indicators should be checked. Ultimately, hospitals need to implement outcome metrics that gauge the utilization of PSI codes or the precision of ‘Y’ or ‘N’ tagging for POA indicators [8, 10]. The Healthcare Quality Evaluation Grant Initiative, a pay-for-performance model, is already making strides towards improving the assessment of POA indicators [23]. However, there is a pressing need to bolster the evaluation framework for PSI codes. Moreover, offering incentives based on the outcomes of these accuracy evaluations could significantly elevate the importance placed on PSI codes and POA indicators in hospital settings.

This study has several limitations. First, the participation of only a select number of hospitals in the PSII, specifically regional public hospitals, may not allow our findings to accurately reflect the full spectrum of healthcare providers in South Korea. Subsequent studies are needed to assess the accuracy of PSI codes and POA indicators across a more diverse array of healthcare institutions, including university hospitals and nursing facilities.

Second, this study’s analysis was confined to verifying the presence of major AE-related diagnosis codes and their respective PSI codes. A more accurate assessment of PSI codes and POA indicators will require a concordance check between more specific AE types and corresponding PSI codes. Given these limitations, the likelihood of overestimating the accuracy of PSI codes reported in this study cannot be ruled out. Follow-up research should focus on examining the presence of PSI codes based on more specific characteristics of AEs.

Finally, variables related to accuracy of the PSI codes and POA indicators could not be identified. Although Pearson’s correlation coefficient was used, statistically significant results were not confirmed. While the accuracy of PSI codes and POA indicators is expected to be affected by individual medical institutions’ coding practices and audit procedures, such information was not available in this study. In future research, it is necessary to explore factors that affect the accuracy of PSI codes and POA indicators.

## Conclusion

In this study, we analysed diagnosis codes and POA indicators for patients with confirmed AEs using PSII data to assess their accuracy. Ensuring the precision of PSI codes and POA indicators is essential to effectively leverage administrative data to gauge patient safety status. However, our findings indicate that the accuracy of PSI codes and POA indicators in South Korean hospitals is not at an optimal level. Furthermore, we observed considerable variability in accuracy among the hospitals involved in the PSII. This variability underscores the need for targeted efforts to enhance the accuracy of PSI codes and POA indicators in individual hospitals and for more rigorous evaluations at the national level. The lack of comprehensive research on the accuracy of PSI codes and POA indicators across many countries worldwide, including South Korea, could be mitigated by undertaking similar studies in other countries using the methodology employed in this study.

## Data Availability

The datasets used and/or analysed during the current study are available from the corresponding author on reasonable request.

## Abbreviations

PSIs: Patient safety incidents
KCD-7: Korean Standard Classification of Disease, Version 7
POA: Present-on-admission; PSII: Patient Safety Incidents Inquiry
